# Hypoglycemic Agents Increase Regulatory Factor X1 to Inhibit Cancer Cell Behaviour in Human Glioblastoma Cells

**DOI:** 10.1111/jcmm.70260

**Published:** 2024-12-05

**Authors:** Weiran Shan, Kendrick Zuo, Zhiyi Zuo

**Affiliations:** ^1^ Department of Anesthesiology University of Virginia Charlottesville Virginia USA; ^2^ Kenneth P. Dietrich School of Arts and Sciences University of Pittsburgh Pittsburgh Pennsylvania USA

**Keywords:** human glioblastoma cells, metalloproteinase 2, regulatory factor X1, thiazolidinediones, WY‐14643

## Abstract

Glioblastoma multiforme is a deadly brain tumour in humans. We have shown that regulatory factor X1 (RFX1), a transcription factor, inhibits the proliferation, migration and invasion of human glioblastoma cells. This study was designed to identify the existing medications that could increase RFX1 in human glioblastoma cells and to determine whether these medications could inhibit the cancer cell behaviours. A bioinformatics approach was used to identify the medications that increased RFX1. The effects of these medications on human glioblastoma cell proliferation, migration and invasion were assayed under cell culture and mouse brain xenograft conditions. Pioglitazone, rosiglitazone and WY‐14643 increased RFX1 based on bioinformatics prediction and Western blotting data. These hypoglycemic agents reduced the proliferation, migration and invasion of human glioblastoma cell cultures. These agents reduced metalloproteinase 2 (MMP2) activity in the culture medium. Silencing RFX1 attenuated hypoglycemic agent‐induced inhibition of cancer cell behaviours and MMP2 activity. Pioglitazone reduced the xenograft tumour volume and migration distance of U87 human glioblastoma cells in the mouse brain. RFX1‐siRNA attenuated these effects. Our results provide additional evidence for RFX1 as a therapeutic target for human glioblastoma and suggest that pioglitazone, rosiglitazone and WY‐14643 inhibit cancer cell behaviour of human glioblastoma cells via upregulating RFX1.

## 1 Introduction

Glioblastoma is an incurable brain tumour in humans with a very poor outcome [1]. One of the major difficulties in treating glioblastoma is its resistance to chemotherapy and radiotherapy [1, 2]. It has been shown that clusters of differentiation (CD) 44 is increased in human glioblastoma tissues, which contributes to the development of resistance to chemotherapy [3]. CD44 can interact with growth factor receptors to enhance cell survival, migration and invasion [4]. Our study has shown that regulatory factor X1 (RFX1) is a tumour‐suppressive transcription factor that can downregulate the expression of CD44 and growth factors [5, 6]. Overexpression of RFX1 in human glioblastoma cells increases apoptosis and reduces cell proliferation, migration and invasion in cell culture or xenograft models [6]. However, RFX1 is silenced in glioma cells [7]. Thus, increasing RFX1 may be an effective approach to inhibit cancer cell behaviours, such as cell proliferation, migration and invasion. To provide evidence to support this approach, we used a bioinformatics‐based drug repositioning approach [8] to identify medications that could increase RFX1 expression. Three hypoglycemic agents, pioglitazone, rosiglitazone and WY‐14643, were identified to have such an effect.

Pioglitazone and rosiglitazone are thiazolidinediones that activate peroxisome proliferator‐activated receptor (PPAR) γ [9]. WY‐14643 is an agonist of PPARα [10]. PPARs are intracellular receptors, whose activation can induce the expression of many genes [11]. Thiazolidinediones have been found to inhibit cancer cell behaviour in various tumour cells via PPARγ‐dependent and PPARγ‐independent mechanisms, such as a decrease of metalloproteinase 2 (MMP2) activity [9, 12, 13]. However, the effects of thiazolidinediones on glioblastoma cells and the role of RFX1 in the pharmacological effects of thiazolidinediones are not known. Much less is known about the effects of WY‐14643 on cancer cells. Thus, this study is designed to determine whether pioglitazone, rosiglitazone and WY‐14643 can reduce cancer cell behaviour of human glioblastoma via increasing RFX1. This determination was performed in cell cultures and xenografts in the mouse brain.

## 2 Methods and Materials

By using the bioinformatics‐based drug‐repositioning approach reported before [8], three hypoglycemic drugs, pioglitazone, rosiglitazone and WY‐14643, were identified as candidate drugs to increase RFX1 expression.

### 2.1 3‐(4,5‐Dimethylthiazol‐2‐Yl)‐2,5‐Diphenyltetrazolium Bromide (MTT) Assay

Cell proliferation was measured by MTT assay. Human glioblastoma U251 (3000 cells/well) and U87 (5000 cells/well) cells were seeded on 96‐well plates overnight. Pioglitazone (catalogue number: E6910; Sigma, St. Louis, MO, USA), rosiglitazone (catalogue number: R2408; Sigma) and WY‐14643 (catalogue number: C7081; Sigma) were added to these cells to make the final concentrations of 5, 10, 15, 20, 25 and 30 μM for 72 h before MTT assay. MTT (Catalogue number: CT02; EMD Millipore, Billerica, MA, USA) solution was added and incubated with cells for 4 h at 37°C. Formazan was dissolved in isopropanol with 0.04 N HCl to measure the absorbance at 570 nm (630 nm as a reference wavelength) by a microplate reader (model 680; Bio‐Rad Laboratories, Hercules, CA, USA). The OD values were normalised with untreated cells. At least 3 independent experiments with three repeated wells for each treatment condition were carried out for each condition.

### 2.2 RFX1‐siRNA

The combination of RFX1‐siRNA‐1 and RFX1‐siRNA‐2 was used to knockdown the RFX1 expression in U251 and U87 cells as we did before [6]. Briefly, cells were plated on six‐well or twelve‐well plates for 24 h before transfection. RFX1‐siRNAs or non‐targeting (NT)‐siRNA were transfected into cells with Lipofectamine 2000 and Opti‐Mem I (Invitrogen, Eugene, ON, USA) to a final concentration of 4 pM. Cells were incubated with siRNA for 72 h and then used for subsequent experiments. The RFX1‐siRNA sequences were as follows:

RFX1‐siRNA‐1 (sense: 5’‐GGGCAACUCCAAGUACCACUACUAU‐3′; antisense: 5′‐AUAGUAGUGGUACUUGGAGUUGCCC‐3′) and RFX1‐siRNA‐2 (sense: 5′‐UGGAAAUCCUCAUUCCCGACGUGCU‐3′; antisense: 5′‐AGCACGUCGGGAAUGAGGAUUUCCA‐3′).

NT‐siRNA sequences were as follows: 5′‐AAATGTACTGCGCGTGGAGAC‐3′ and 5′‐GTCTCCACGCGCAGTACATTT‐3′.

### 2.3 Western Blotting

The expression of RFX1 was analysed by Western blotting analysis as we did before [6, 14] after being treated with pioglitazone, rosiglitazone and WY‐14643 for 72 h and with or without the transfection of RFX1‐siRNA. The cells were harvested and lysed in RIPA buffer (Thermo Fisher Scientific, Waltham, MA, USA) containing a protease inhibitor cocktail (Sigma). Twenty micrograms of proteins in each lane was separated on 10% gel and then transferred to polyvinylidene difluoride membranes. Primary antibodies used were as follows: mouse monoclonal anti‐RFX1 (H‐2) antibody (1:1000 dilution, catalogue number: sc‐376041; Santa Cruz Biotechnology) and rabbit polyclonal anti‐glyceraldehyde 3‐phosphate dehydrogenase antibody (GAPDH) (1:5000 dilution, catalogue number: G9545; Sigma). Secondary antibodies were goat antirabbit antibody conjugated with horseradish peroxidase (1:5000 dilution; Santa Cruz Biotechnology) and goat antimouse antibody conjugated with horse radish peroxidase (1:5000 dilution; Santa Cruz Biotechnology). Protein bands were analysed by Genesnap version 7.08 and quantified by Genetools version 4.01. The relative protein expression of RFX1 was normalised by GAPDH of the same sample. The results of various conditions were normalised by those of the control group. At least three independent experiments and 8 samples of each group were analysed.

### 2.4 Wound Healing and Matrigel Invasion Assay

U251 and U87 cells transfected with or without RFX1‐siRNA were cultured in six‐well plates and treated with pioglitazone, rosiglitazone or WY‐14643 for 48 h. For the wound healing assay, a straight scratch was made with a 200 μL pipette tip. The culture medium containing cell debris was aspirated away and replaced by fresh serum‐free medium. The wound healing was assessed at 0 and 24 h after the scratch using a light microscope at 100 × magnification. The sizes and lengths of the area without cells were measured by Image Pro (National Institutes of Health, Bethesda, MD, USA). The size of the area was divided by the lengths of the area to calculate the average distance that had not been covered by cells. The distance migrated at 24 h after the wounding was then calculated.

For matrigel invasion assay, after being treated with pioglitazone, rosiglitazone and WY‐14643 for 48 h, U251 (1×10^5^) and U87 (1.5×10^5^) cells were suspended in serum‐free medium and transferred to the upper chamber of 24‐well plates with 8.0 μm membrane (catalogue number: 354578; Corning, NY, USA) and a coat of Matrigel Matrix (catalogue number: 354234; Corning). The lower chamber was filled with 600 μL complete medium for U251 or U87 cells. Twenty‐four hours later, cells on the upper membrane surface were removed. Those cells that migrated to the lower side of the membrane were fixed and stained with 0.1% crystal violet. Invasive cells were counted in five randomly chosen fields under a microscope at 100 × magnification in a blind fashion, and the average number of cells in these fields was calculated. At least three independent experiments and 8 samples of each group were conducted.

### 2.5 Colony‐Forming Assay

U251 and U87 cells transfected with or without RFX1‐siRNA were seeded at 100 cells/well into 6‐well plates. They were then treated with or without pioglitazone, rosiglitazone and WY‐14643 (25 μM) for 12 days. Medium was changed every 3 days. Cells were then fixed and stained with 1% crystal violet for 30 min. Colonies with more than 50 cells were counted. The number of colonies in the number of seeded cells in percentage was calculated to reflect colony‐forming efficiency.

### 2.6 Measurement of Enzymatic Activity of MMP2

U251 and U87 cells transfected with or without RFX1‐siRNA were cultured in 6‐well plates. After being treated with pioglitazone, rosiglitazone and WY‐14643 for 72 h, the supernatant of the culture medium was collected for MMP2 activity assay by using Novex Zymogram gels (catalogue number: ZY00100BOX; Thermo Fisher Scientific) as we did before [15, 16]. Gels were scanned by a Bio‐Rad GS 800 densitometer. MMP‐2 activity was quantified by measuring the volume of the band with ImageJ (National Institutes of Health, MD, USA).

### 2.7 RFX1 Promoter Activity Assay

Gene on chromosome 19 from 14,118,605 to 14,119,089 containing the promoter region of RFX1 (5′‐TCACCCCGGGCCCCGCCCCCGCGCGTGACGTAACTAGGGTGGGTAGCAACAGTTGCCCC‐3′) was amplified and engineered into the vector pGL3‐Luc (dual‐luciferase reporter assay system, E1910; Promega, Madison, WI, USA) to generate pGL3‐RFX1 as we demonstrated before [5, 6]. The primers used to amplify the promoter region that contained KpnI and HidIII enzymatic sites (letters in italic format) were as follows:

Sense primer: 5’‐TCC*GGTACC*GAGTCTTCCTTTCTCGCCTTTT‐3′ and antisense primer: 5’‐GGC*AAGCTT*GCCTCCGTTTCCCTCACC‐3’.

Constructed plasmid was sequenced to confirm the accuracy of DNA sequences before they were transfected into U87 cells. The cells were then treated with or without hypoglycemic agents at 15 or 25 μM for luciferase activity assay. The Renilla‐luciferase expression plasmid was co‐transfected as the internal control. The experiment was conducted at least three times.

### 2.8 Implantation of U87 Cell into Mouse Brain

Five microliters of U87 cells (about 100,000 cells) were injected into the posterior striatum of Hsd:Athymic Nude‐Foxn1^nu^ (Envigo, Dublin, VA, USA) mice. Injection site coordinates were 2.5 mm from the middle line, −1 mm from Bregma and at a depth of 3 mm from the skull. The local wound around the hole was infiltrated with 0.2 mg/kg bupivacaine to relieve the pain for the first 24 h post‐operatively. Pioglitazone was administrated by oral gavage at a dosage of 5 mg/kg once a day for 8 days with the first dose at 3 days after cell injection. Five groups of animals were studied: blank control (8 mice), blank control plus pioglitazone (blank+Pio, 8 mice), RFX1‐siRNA only (6 mice), NT‐siRNA plus pioglitazone (NT‐siRNA+Pio, 8 mice) and RFX1‐siRNA plus pioglitazone (RFX1‐siRNA+Pio, 7 mice).

### 2.9 Immunofluorescent Staining and Determination of Tumour Volume and Migration Distance in Mouse Brain

Two weeks after the injection of U87 cells, the mouse brain was harvested. Whole brains were serially sectioned at a thickness of 10 μm. Six coronary sections from the injection site (3 sections anterior and 3 sections posterior to the injection site) were used for immunofluorescent staining. Antihuman nucleus antibody clone 3E1.3 (1:200 dilute, catalogue number: MAB4383; Millipore, Billerica, MA, USA) was used for staining U87 cell nuclei. Secondary antibody was donkey antimouse IgG conjugated with Alexa Fluor 594 (1:200 dilution, catalogue number: A‐21203; Invitrogen). Sections were counterstained with Hoechst 33342 (1:1000 dilution, catalogue number: 62249; Thermo Fisher Scientific, Pittsburgh, PA) for 5 min, rinsed and mounted with Vectashield mounting medium (catalogue number: H‐1000; Vector Laboratories, Burlingame, CA). Images were acquired with a fluorescent Olympus DP70 microscope (Olympus Corporation, Tokyo, Japan).

Tumour volumes and the invasion distance of cells were assessed as we described before [17]. These measures were performed by using ImageJ by a blinded observer. Tumour area was manually outlined by a freehand tool and then measured in μm^2^. The area was then multiplied by the section thickness (10 μm) to obtain the tumour volume in the section. Volumes of all sections (6 sections/mouse) were added together to achieve the total volume of each tumour. Invasion distance of cells from the edge of the main tumour bulk was measured by using ImageJ. The invasion distances of all cells stained by the antihuman nucleus antibody in the 6 sections from each mouse were measured. The longest invasion distance of U87 cells in each mouse was then identified.

### 2.10 Statistical Analysis

Statistical analyses were performed using IBM SPSS statistics 27. Results were presented as means ± S.D. when the data were in normal distribution or as median and 95% confidential interval if the data were non‐normal distribution. The data were analysed by one‐way analysis of variance followed by Tukey's test if the data were normally distributed or by Kruskal–Wallis one‐way ANOVA test followed by Dunn's post hoc tests if the data were not normally distributed. Differences were considered significant at *p* < 0.05 based on two‐tailed hypothesis testing.

## 3 Results

### 3.1 Pioglitazone, Rosiglitazone and WY‐14643 Increased RFX1 Expression in Human Glioblastoma Cells

**FIGURE 1:**
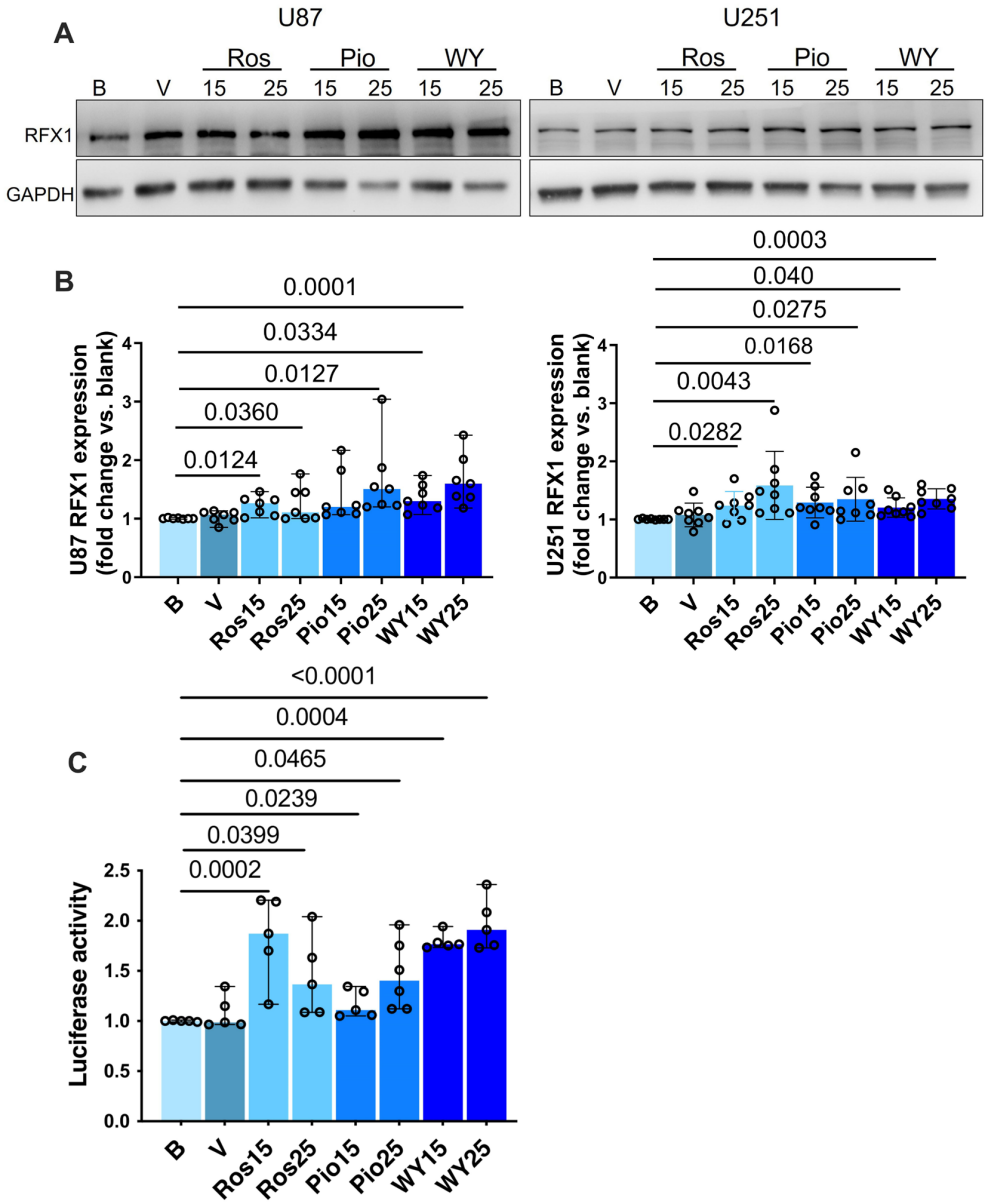
Pioglitazone, rosiglitazone and WY‐14643 increased the expression of RFX1 protein and the activity of presumable RFX1 promoter. U87 or U251 cells were incubated with 15 or 25 μM pioglitazone, rosiglitazone and WY‐14643 for 3 days. Cells were harvested for Western blotting. In another experiment, U87 cells were transfected with a plasmid containing code for luciferase, whose expression was under the control of a presumable RFX1 promoter. Three days after the transfection, U87 cells were incubated with 15 or 25 μM pioglitazone, rosiglitazone and WY‐14643 for 48 h for luciferase activity assay. (A) Representative images of Western blotting. (B) Quantitative results of Western blotting analysis. (C) Luciferase activity results. Results are median and 95% confidence interval (*n* = 7 to 8 for panel B and 5 to 6 for panel C) with the presence of the values of the individual experiment. B, blank control; Pio, pioglitazone; Ros, rosiglitazone; V, vehicle; WY, WY‐14643.

Two human glioblastoma cell lines, U87 and U251 cells, were used in the study. The incubation of U87 cells with 15 or 25 μM rosiglitazone or WY‐14643 or 25 μM pioglitazone for 72 h increased the RFX1 protein. Similarly, the incubation of 15 or 25 μM pioglitazone, rosiglitazone and WY‐14643 increased the RFX1 protein in the U251 cells (Figure 1A,B). These results confirmed the prediction using the bioinformatics‐based approach that pioglitazone, rosiglitazone and WY‐14643 increase RFX1 expression in the glioblastoma cells.

Pioglitazone, rosiglitazone and WY‐14643 increased the activity of luciferase, whose expression is controlled by the presumed RFX1 promoter region in the U87 cells (Figure 1C). These results suggest that pioglitazone, rosiglitazone and WY‐14643 increase the activity of the RFX1 promoter, indicating that the increased expression of the RFX1 protein by these hypoglycemic agents is due to the increased transcription of the *rfx1* gene.

### 3.2 Pioglitazone and Rosiglitazone Inhibited Cancer Cell Behaviour of Human Glioblastoma Cells

**FIGURE 2:**
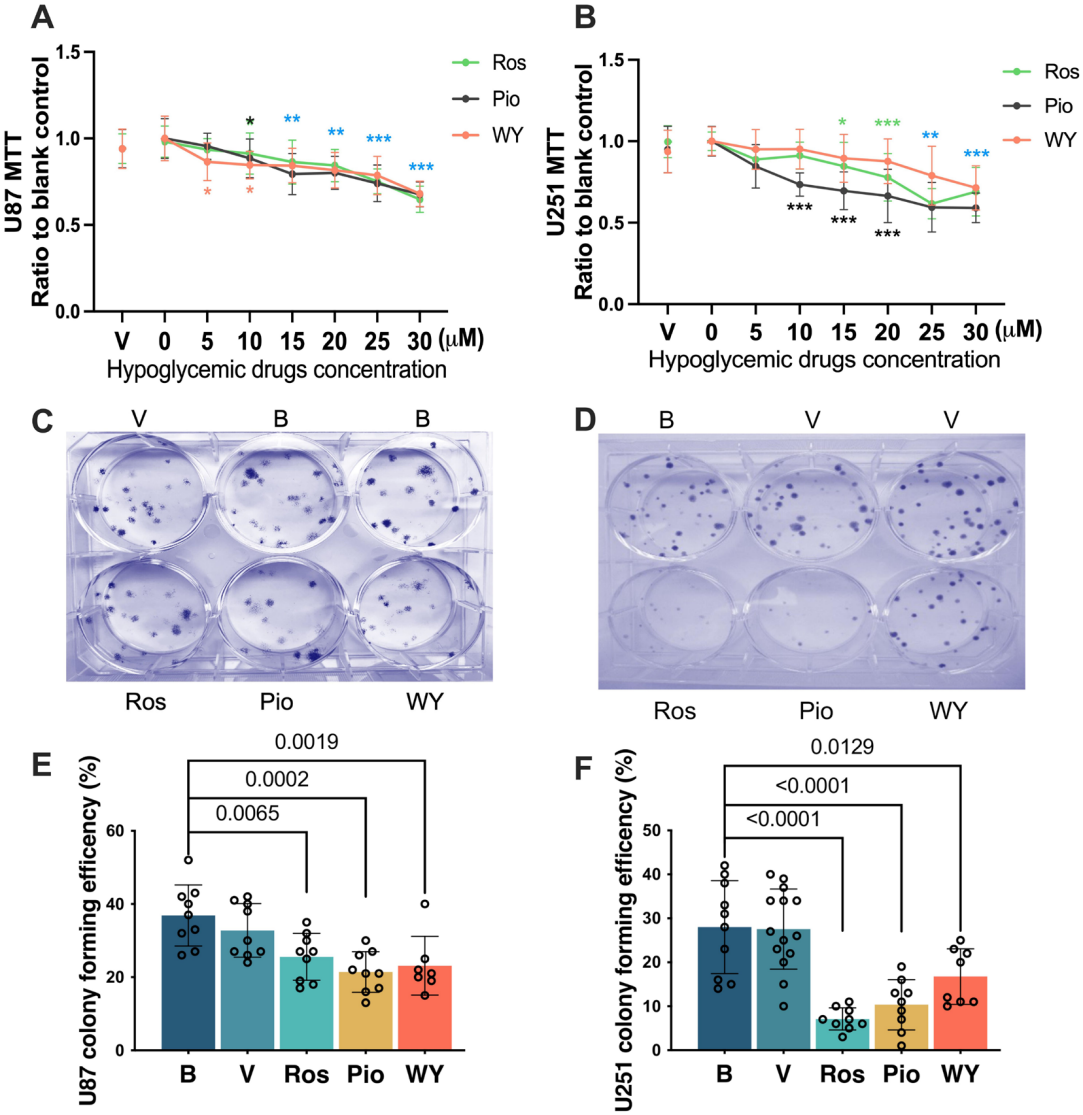
Pioglitazone, rosiglitazone and WY‐14643 reduced the number of cells and cell colonies. U87 or U251 cells were incubated with various concentrations of pioglitazone, rosiglitazone and WY‐14643 for 3 days for MTT assay. In another study, 100 U87 or U251 cells were plated to each well of 6‐well plates. The colonies formed were counted 12 days later. (A, B) Quantitative results of the MTT assay. Results are mean ± S.D. (*n* = 8 to 18). *p < 0.05, **p < 0.01, ***p < 0.001 compared with the corresponding results of blank control. The colour of * indicates the comparison of conditions in each medication. (C, D) Representative images of colony formation. (E, F) Quantitative results of colony formation. Results are mean ± S.D. (*n* = 7 to 9 for panel E and 8 to 14 for panel F) with the presence of the values of the individual experiment. B, blank control; Pio, pioglitazone; Ros, rosiglitazone; V, vehicle; WY, WY‐14643.

Pioglitazone, rosiglitazone and WY‐14643 dose‐dependently decreased the value of MTT assay in the U87 and U251 cells (Figure 2A,B), suggesting that the number of cells or the activity of the NADPH‐dependent cellular oxidoreductases decreased with increased concentrations of pioglitazone, rosiglitazone and WY‐14643. Similarly, incubation with 25 μM pioglitazone, rosiglitazone and WY‐14643 for 12 days reduced the number of colonies formed by U87 and U251 cells (Figure 2C–F). These results suggest that the proliferation and survival of these two human glioblastoma cell lines are reduced by the hypoglycemic agents.

**FIGURE 3:**
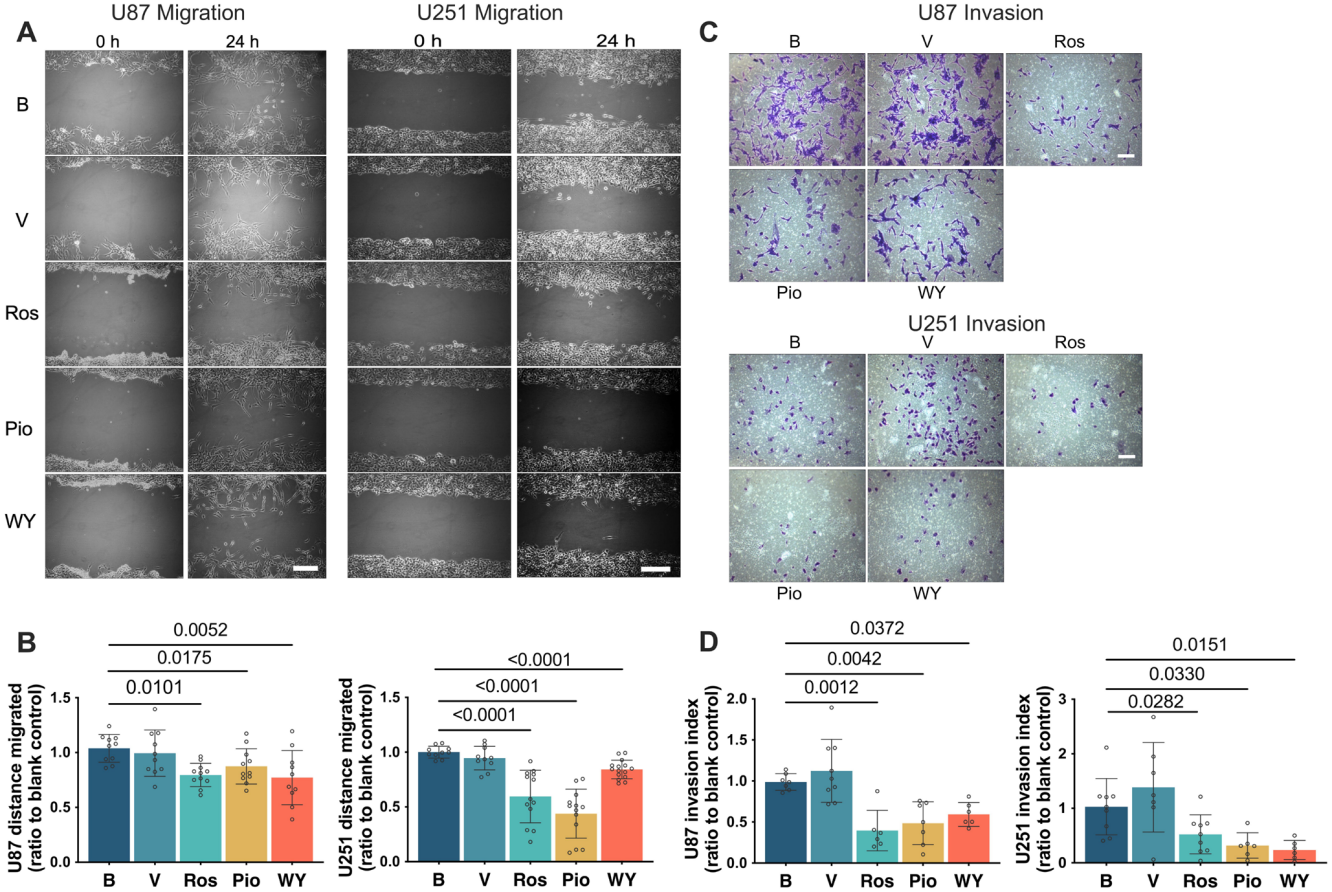
Pioglitazone, rosiglitazone and WY‐14643 reduced the migration and invasion of human glioblastoma cells. Confluent U87 and U251 cell cultures were incubated with 25 μM pioglitazone, rosiglitazone and WY‐14643 for 2 days and then received a scratch of a 200 μL pipette tip. The migration of cells through the scratch was evaluated one day later. In another experiment, U87 and U251 cell cultures were incubated with 25 μM pioglitazone, rosiglitazone and WY‐14643 for 2 days and then transferred to matrigel invasion assay plates. The number of cells invading into the lower chamber of the plates was counted 24 h later. (A) Representative images of migration experiments. Scale bar = 200 μm. (B) Quantitative results of migration experiments. (C) Representative images of invasion experiments. Scale bar = 200 μm. (D) Quantitative results of invasion experiments. Results are mean ± S.D. (*n* = 10 to 15 for panel B and *n* = 6 to 9 for panel D) with the presence of the values of the individual experiment. B, blank control; Pio, pioglitazone; Ros, rosiglitazone; V, vehicle; WY, WY‐14643.

Pioglitazone, rosiglitazone and WY‐14643 reduced the migration distance of U87 and U251 cells (Figure 3A,B). Similarly, these agents decreased the number of cells that penetrated from the upper chamber to the lower chamber in the matrigel invasion assay (Figure 3C,D). These results suggest that pioglitazone, rosiglitazone and WY‐14643 reduce the migration and invasion of human glioblastoma cells.

### 3.3 Pioglitazone, Rosiglitazone and WY‐14643 Reduced MMP2 Activity via Increasing RFX1 in Human Glioblastoma Cells

**FIGURE 4:**
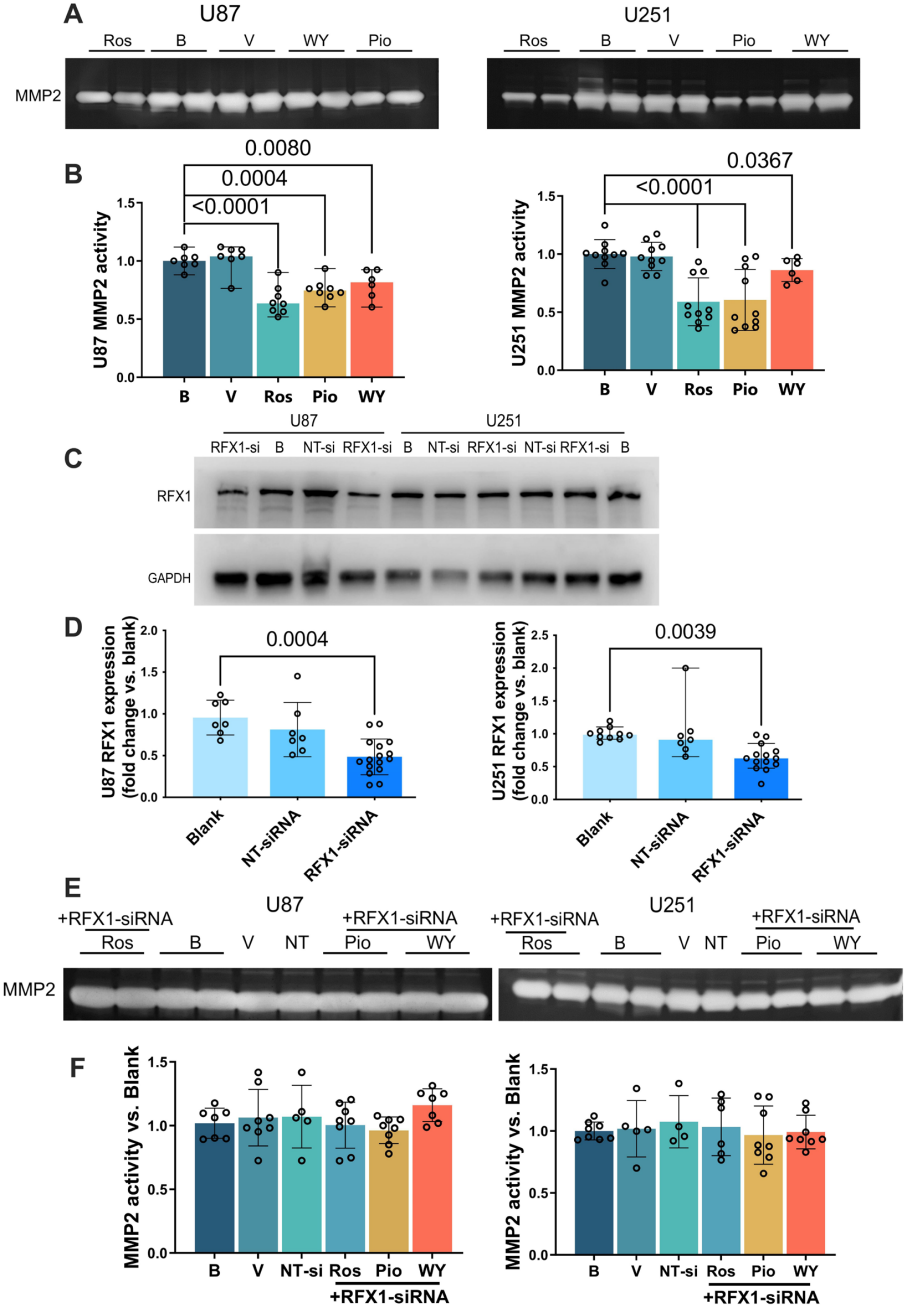
Pioglitazone, rosiglitazone and WY‐14643 reduced MMP2 activity via RFX1. U87 and U251 cells were incubated with 25 μM pioglitazone, rosiglitazone and WY‐14643 for 3 days. The culture medium was harvested for zymogram assay. In another experiment, U87 and U251 cells were transfected with RFX1‐siRNA or non‐targeting (NT) siRNA. Three days later, the cells were harvested for Western blotting. In the third experiment, U87 and U251 cells were transfected with RFX1‐siRNA or NT‐siRNA for 72 h and then incubated with 25 μM pioglitazone, rosiglitazone and WY‐14643 for 3 days. The culture medium was harvested for MMP2 activity assay. (A) Representative images of zymogram assay. (B) Quantitative results of MMP2 activity. (C) Representative images of Western blotting. (D) Quantitative results of Western blotting. (E) Representative images of zymogram assay. (F) Quantitative results of MMP2 activity. Results are median and 95% confidence interval (*n* = 6 to 10 for panel B, 7 to 16 for panel D) or mean ± S.D. (*n* = 4 to 8 for panel F) with the presence of the values of the individual experiment. B, blank control; Pio, pioglitazone; Ros, rosiglitazone; V, vehicle; WY, WY‐14643.

Pioglitazone, rosiglitazone and WY‐14643 reduced the activity of MMP2 in the culture medium of U87 and U251 cells (Figure 4A,B). However, this reduction disappeared after RFX1 expression was silenced by RFX1‐siRNA (Figure 4C–F). These results suggest that the decrease of MMP2 activity by the hypoglycemic agents is via RFX1.

### 3.4 Increasing RFX1 Mediated Hypoglycemic Agent‐Induced Reduction of Migration and Invasion of Human Glioblastoma Cells

**FIGURE 5:**
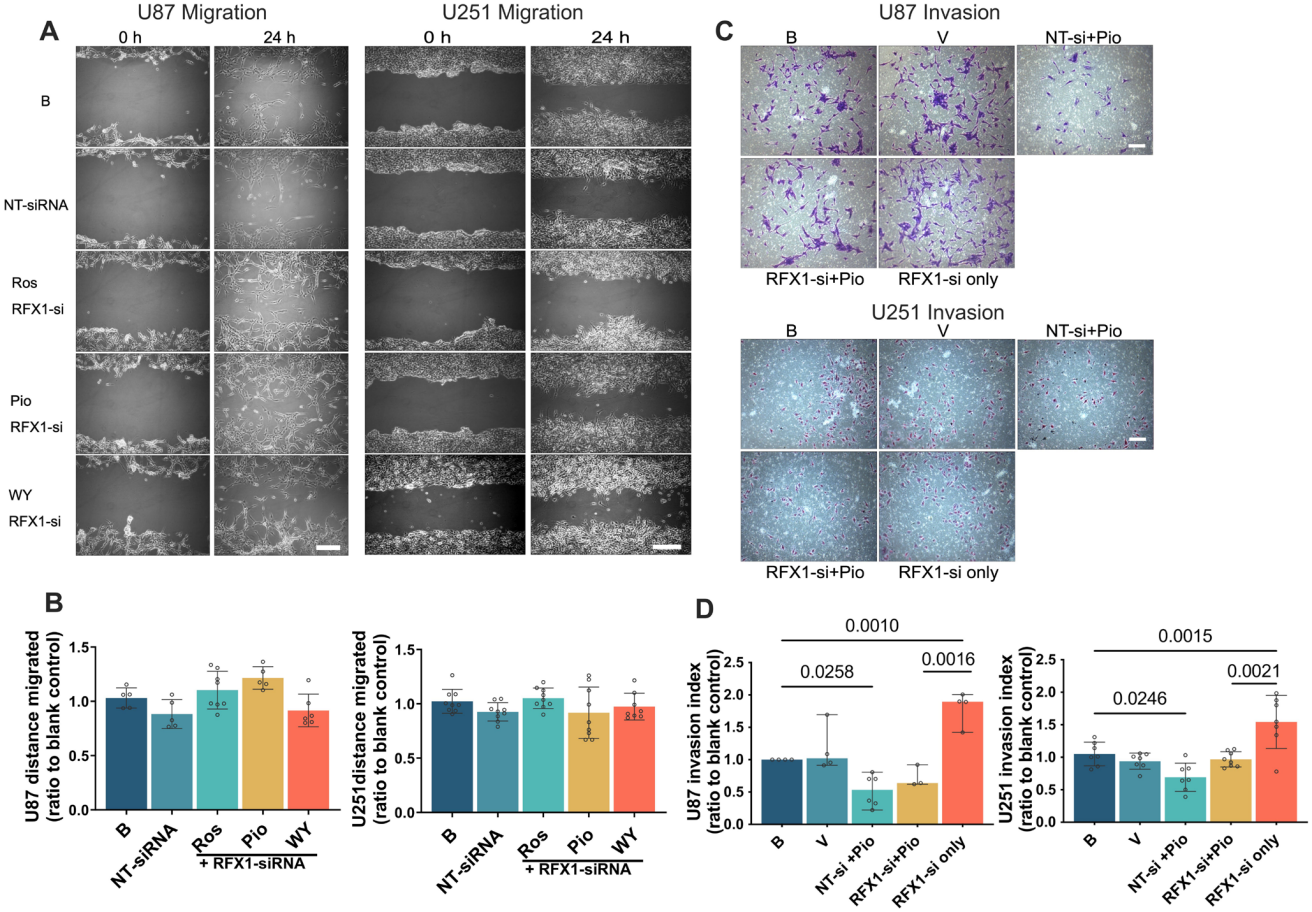
RFX1 downregulation attenuated the reduction of migration and invasion of human glioblastoma cells. Confluent U87 and U251 cell cultures were transfected with RFX1‐siRNA or non‐targeting (NT) siRNA and then incubated with 25 μM pioglitazone, rosiglitazone and WY‐14643 for 2 days. The cell culture then received a scratch of a 200 μL pipette tip. The migration of cells through the scratch was evaluated one day later. In another experiment, U87 and U251 cell cultures were transfected with RFX1‐siRNA or non‐targeting (NT) siRNA and then incubated with 25 μM pioglitazone for 2 days. They were then transferred to matrigel invasion assay plates. The number of cells invading into the lower chamber of the plates was counted 24 h later. (A) Representative images of migration experiments. Scale bar = 200 μm. (B) Quantitative results of migration experiments. (C) Representative images of invasion experiments. Scale bar = 200 μm. (D) Quantitative results of invasion experiments. Results are mean ± S.D. (*n* = 5 to 9 for panel B) or median and 95% confidence interval (*n* = 3 to 8 for panel D) with the presence of the values of the individual experiment. B, blank control; Pio, pioglitazone; Ros, rosiglitazone; V, vehicle; WY, WY‐14643.

The migration of U87 and U251 cells was not reduced by pioglitazone, rosiglitazone and WY‐14643 in the presence of RFX1‐siRNA (Figure 5A,B). In the matrigel invasion assay, the number of U87 or U251 cells penetrated from the upper chamber to the lower chamber decreased with pioglitazone in the presence of NT‐siRNA but was not affected by pioglitazone in the presence of RFX1‐siRNA compared to control condition. RFX1‐siRNA increased the number of U87 or U251 cells that penetrated from the upper chamber to the lower chamber (Figure 5C,D). These results suggest that hypoglycemic agents reduce migration and invasion of human glioblastoma cells via increasing RFX1.

### 3.5 Pioglitazone Inhibited Growth and Migration of Human Glioblastoma Xenograft in Mouse Brain via RFX1

**FIGURE 6:**
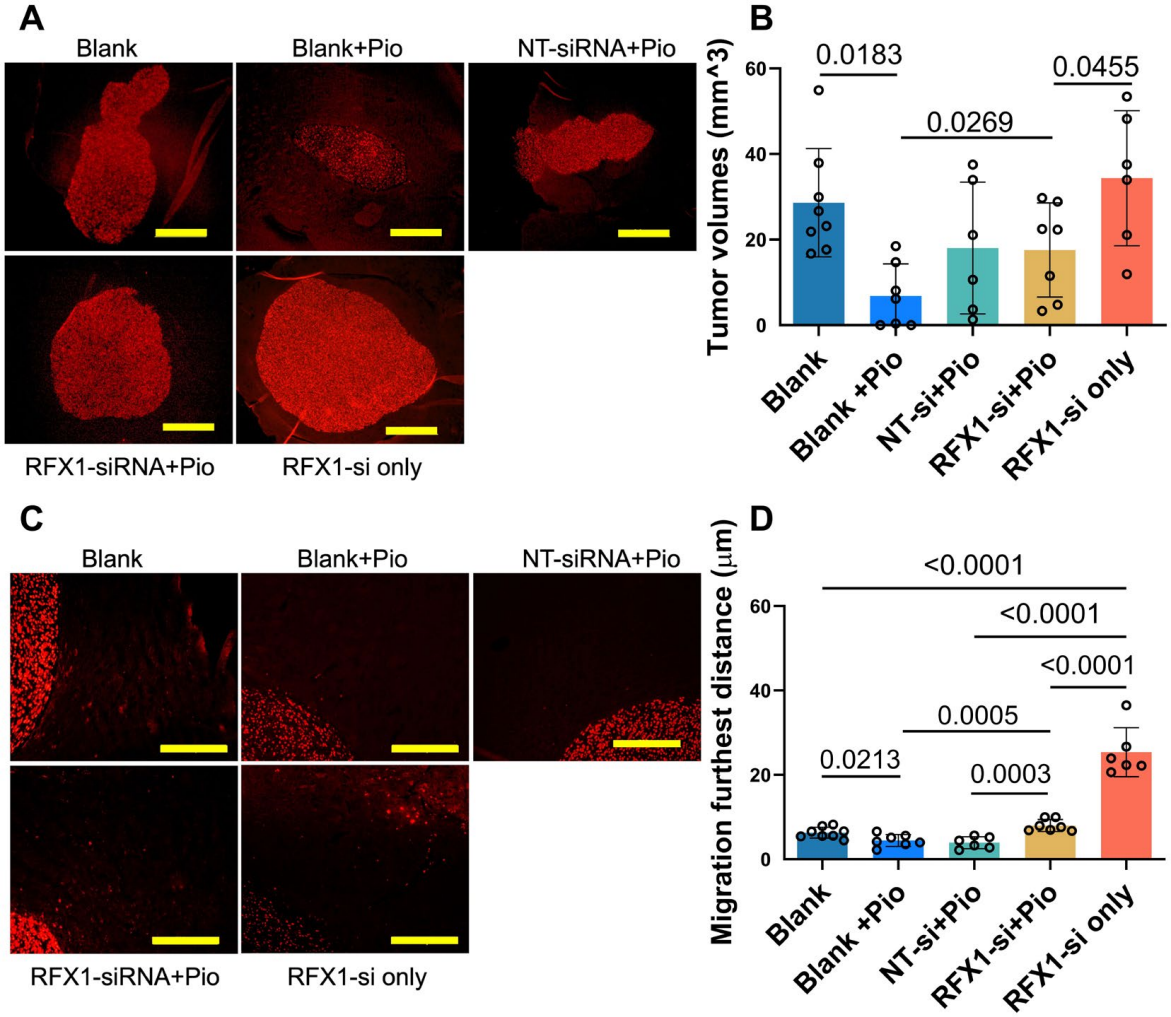
Pioglitazone reduced xenograft tumour volume and invasion of U87 cells via increasing RFX1. U87 cells transfected with RFX1‐siRNA or NT‐siRNA were implanted into the striatum of nude mice. Three days later, some mice received 5 mg/kg/day pioglitazone for 8 days. Mice were sacrificed 12 days after the implantation of cells for the determination of tumour volume and the longest distance of U87 cells invaded into the brain tissues from the original tumour. (A) Representative images of formed brain tumours. Scale bar = 2.0 mm. (B) Quantitative results of brain tumour volume. (C) Representative images of formed brain tumours and small cell masses. Scale bar = 500 μm. (D) Quantitative results of the longest invasion distance. Results are mean ± S.D. (*n* = 6 to 8 for panels B and D) with the presence of the values of the individual experiment. NT‐si, NT‐siRNA ; Pio, Pioglitazone ; RFX1‐si, RFX1‐siRNA.

To determine the effects of pioglitazone on human glioblastoma cells under in vivo conditions, these cells were injected into mouse striatum. Pioglitazone reduced the tumour volume. RFX1‐siRNA but not NT‐siRNA attenuated this reduction (Figure 6A,B). Similarly, pioglitazone reduced the migration distance of U87 cells in the brain. The migration distance of the cells was increased in the presence of pioglitazone plus RFX1‐siRNA (compared with pioglitazone alone or pioglitazone plus NT‐siRNA) or RFX1‐siRNA alone (compared with blank control) (Figure 6C,D). These results suggest a role of RFX1 in the effects of pioglitazone on U87 cell proliferation, survival and migration in the mouse brain.

## 4 Discussion

We have shown that RFX1 is a suppressive transcription factor to inhibit the migration, proliferation and invasion of human glioblastoma cells [6]. As a first step to use this finding for translational research, we used a bioinformatics approach to identify medications that can increase RFX1 expression. Three hypoglycemic agents were identified to have such an effect. Our Western blotting studies have confirmed that these agents increase the RFX1 protein in human glioblastoma cells. Importantly, these agents inhibit the migration, proliferation, survival and invasion of human glioblastoma cells, providing additional evidence for the role of RFX1 in regulating the cancer cell behaviour of glioblastoma cells.

The role of RFX1 in tumour biology has been supported by multiple lines of evidence [18]. First, cancer tissues have a lower expression of RFX1 than the adjacent non‐cancer tissues [19]. Cancer tissues with poorer grades and types have a lower expression of RFX1 [20]. RFX1 reduces the expression of multiple genes that are oncogenic or enhance tumour growth, such as c‐Myc, transforming growth factor‐β2 and CD44 [5, 6, 18]. Finally, RFX1 reduces chemotherapy resistance and cancer recurrence by regulating the expression of genes, including multidrug resistance genes [18]. Thus, increasing RFX1 expression may be an effective therapeutic approach for many different types of cancers, including human glioblastoma. The mechanisms for RFX1's anticancer effects may be through its regulation of the expression of many genes that are involved in tumour genesis and cancer cell biology.

One of the novel findings in this study is that RFX1 can regulate MMP2 activity. Pioglitazone, rosiglitazone and WY‐14643 reduced MMP2 activity. Silencing RFX1 blocked this reduction. Since these hypoglycemic agents increased RFX1 expression, our results suggest that the reduced MMP2 activity by these agents is due to their effects on RFX1 expression. RFX1 regulates the expression of growth factors and multiple intracellular signalling molecules [5, 6], which may modulate the expression of MMP2. For example, Akt can increase MMP2 expression [21]. Overexpression of RFX1 reduces Akt [6]. Thus, hypoglycemic agents can reduce MMP2 activity and expression via these effects of RFX1 on intracellular signalling molecules. In this context, intracellular calcium is an important signalling molecule. Reducing calcium influx inhibits MMP2 expression and activity [22, 23]. It is unclear yet whether intracellular calcium plays a role in the effects of RFX1 on MMP2. MMP2 can break the extracellular matrix [15], which facilitates the migration and invasion of cancer cells. Thus, the reduced MMP2 activity may contribute to the decrease of migration and invasion caused by pioglitazone, rosiglitazone and WY‐14643 in the glioblastoma cells under in vitro and in vivo conditions. Consistent with this possibility, inhibiting MMP2 activity has been considered as a PPARγ‐independent mechanism for thiazolidinediones to reduce invasiveness of pancreatic cancer cells [12].

Thiazolidinediones have been shown to inhibit cancer cell behaviours in multiple types of cancer cells [9]. The effects include inhibition of cell growth, invasion and migration and induction of apoptosis. PPARγ‐dependent and PPARγ‐independent mechanisms have been shown for these effects [9, 12, 13]. WY‐14643 may reduce angiogenesis to reduce cancer growth [24]. Our study provides evidence to suggest that thiazolidinediones and WY‐14643 can increase RFX1 expression to inhibit cancer cell behaviour, a novel mechanism for their anticancer effects. The mechanism for this increased expression appears to increase the transcription of RFX1 because these agents increased the promoter activity of the *rfx1* gene in our study.

Our findings in this study may have clinical implications. Thiazolidinediones are used clinically to treat patients with type 2 diabetes [25]. If their anticancer effects are confirmed in clinical trials, their use to benefit patients with cancer can be rapidly implemented. Interestingly, two retrospective studies have shown survival benefits in patients with diabetes and prostate cancer or diabetes and multiple myeloma, respectively [26, 27]. Clinical trials are needed to test the anticancer effects of thiazolidinediones, including their effects to improve the sensitivity of cancer cells to chemotherapy to determine the new therapeutic use of these agents that are currently used for controlling diabetes. Interestingly, pioglitazone may increase the risk of bladder cancer [28] although no effects have also been reported [29, 30]. Additional studies are needed to clarify this issue (carcinogenesis potential) for the consideration of using this agent to inhibit neuroblastoma cell growth and migration clinically. Obviously, carcinogenesis and cancer cell behaviours are different biological processes. However, the effects on cancer cell survival and growth will affect whether a few cancer cells can become a cancer lesion.

Our study has limitations. First, we have shown that pioglitazone, rosiglitazone and WY‐14643 reduce MMP2 activity by increasing RFX1. It is not clear whether this effect was via decreasing the expression of MMP2 or inhibiting the activation of the enzyme. A previous study has shown that thiazolidinediones reduced the transcription of MMP2 in pancreatic cancer cells [12]. Since RFX1 is a transcription factor, it is possible that RFX1 downregulates the expression of MMP2 to reduce its activity. Future studies will determine the mechanisms for RFX1 to regulate MMP2 activity. We have also not determined the mechanisms for thiazolidinediones and WY‐14643 to reduce the number of human glioblastoma cells and the colonies formed by these cells. However, we have shown that RFX1 can induce apoptosis and inhibit cell proliferation of glioblastoma cells via inhibiting CD44 and related signalling molecules [6]. RFX1 can also inhibit the expression of many growth factors [5]. These mechanisms may contribute to the inhibition of cell proliferation and colony formation by thiazolidinediones and WY‐14643 in this study. Finally, the results of the MTT assay suggest the reduced number of human glioblastoma cells by hypoglycemic agents. Results of MTT assay alone are not sufficient for this suggestion because the method mainly measures the activity of the NADPH‐dependent cellular oxidoreductases [31]. However, the reduced number of human glioblastoma cells by hypoglycemic agents is also supported by the results of colony formation and xenograft assays in this study.

In summary, we have shown that a bioinformatics‐based approach has identified that thiazolidinediones and WY‐14643 can induce RFX1 expression, which is confirmed by assessing the protein expression of RFX1 in human glioblastoma cells. Thiazolidinediones and WY‐14643 inhibit the survival, migration and invasion of glioblastoma cells via increasing RFX1 in these cells under cell culture and xenograft conditions. These results suggest the therapeutic potential of thiazolidinediones for anticancer purposes in addition to their hypoglycemic effects for treating diabetes.

## Author Contributions

**Weiran Shan:** data curation (lead), formal analysis (equal), investigation (lead), methodology (lead), writing – original draft (supporting). **Kendrick Zuo:** data curation (supporting), methodology (supporting). **Zhiyi Zuo:** conceptualization (lead), formal analysis (lead), funding acquisition (lead), investigation (lead), project administration (lead), resources (lead), supervision (lead), validation (lead), writing – original draft (lead), writing – review and editing (lead).

## Ethics Statement

**Approval of the research protocol by an IRB:** Not applicable.

**Informed consent:** Not applicable.

**Registry and registration number of the study/trial:** Not applicable.

**Animal study:** All animal procedures were approved by our Institutional Animal Care and Use Committee, University of Virginia, Charlottesville, VA 229089, USA.

**Consent for publication:** Not applicable.

## Conflicts of Interest

The authors declare no conflicts of interest.

## Data Availability

The data are available upon a reasonable request.
